# c-Rel Deficiency Increases Caspase-4 Expression and Leads to ER Stress and Necrosis in EBV-Transformed Cells

**DOI:** 10.1371/journal.pone.0025467

**Published:** 2011-10-03

**Authors:** Aníbal Valentín-Acevedo, Frank L. Sinquett, Lori R. Covey

**Affiliations:** Department of Cell Biology and Neuroscience, Rutgers University, Piscataway, New Jersey, United States of America; University of Miami, United States of America

## Abstract

LMP1-mediated activation of nuclear factor of kappaB (NF-κB) is critical for the ligand independent proliferation and cell survival of *in vitro* EBV-transformed lymphoblastoid cell lines (LCLs). Previous experiments revealed that a majority of LMP1-dependent responses are regulated by NF-κB. However, the extent that individual NF-κB family members are required for these responses, in particular, c-Rel, whose expression is restricted to mature hematopoietic cells, remains unclear. Here we report that low c-Rel expression in LCLs derived from a patient with hyper-IgM syndrome (Pt1), resulted in defects in proliferation and cell survival. In contrast to studies that associated loss of NF-κB with increased apoptosis, Pt1 LCLs failed to initiate apoptosis and alternatively underwent autophagy and necrotic cell death. Whereas the proliferation defect appeared linked to a c-Rel-associated decrease in c-myc expression, identified pro-survival and pro-apoptotic targets were expressed at or near control levels consistent with the absence of apoptosis. Ultrastructural examination of Pt1 LCLs revealed a high level of cellular and ER stress that was further supported by gene expression profiling showing the upregulation of several genes involved in stress and inflammation. Apoptosis-independent cell death was accompanied by increased expression of the inflammatory marker, caspase-4. Using gene overexpression and siRNA knockdown we demonstrated that levels of c-Rel directly modulated expression of caspase-4 as well as other ER stress genes. Overall, these findings reveal the importance of c-Rel in maintaining LCL viability and that decreased expression results in ER stress and a default response leading to necrotic cell death.

## Introduction

Constitutive activation of the Rel/NF-κB signal transduction pathway has been associated with a wide range of malignancies in both animals and humans and thus has long constituted a favored target for potential therapeutic intervention [1]. Among the multiple Rel/NF-κB family members, only c-Rel, the cellular counterpart of v-Rel, whose expression leads to aggressive lymphomas in chickens [2], has demonstrated the capacity to malignantly transform cells in culture [3], [4]. Accordingly, gene amplification or persistent activation of c-Rel is detected in many human B cell tumors including diffuse large B cell lymphoma (DLBCL), Hodgkin's lymphoma, follicular B cell lymphoma and mediastinal large B-cell lymphoma [5], [6], [7], [8]. Recent results using siRNA gene silencing found that downregulating c-Rel in a B cell tumor line resulted in a marked increase in apoptotic cell death highlighting the potential for c-Rel as a therapeutic target [9].

Although c-Rel is expressed predominantly in mature lymphoid and myeloid tissues its expression and function has been most fully characterized in B cells where it is required for both antigen-dependent differentiation and late-stage effector functions [10], [11], [12], [13]. Targeted deletion of *c-rel* in B cells elicited primary defects in mitogen- and antigen-induced proliferation as well as a significant loss of antibody production in antigen-responding cells [14], [15]. The proliferative defect was effectively complemented by over-expression of cyclin E, which is induced by the c-Rel-mediated transcription factor E2F3a as well as by Bcl-xL [16], [17]. In addition to proliferative defects, *c-rel*-deficient B cells were highly sensitized to apoptosis through pathways activated by antigen receptor engagement, gamma-irradiation and dexamethasone treatment [18], [19], [20], [21]. In B cells, c-Rel is expressed in response to BCR, CD40 and BAFF-R (BR3) signaling and known regulators include PI3 kinase/Akt [18], [22], [23], [24], Bcl-10 [25], Carma-1 [26], [27], and isoforms of protein kinase C [28]. These signals result in c-Rel expression through autoregulation and by the activation of transcription factors SpiB and PU.1 that bind to specific sites in the c-Rel promoter (reviewed in [29]).

Our interest in the relationship of c-Rel expression to B cell function extends from our characterization of a patient with non-X-linked hyper-IgM syndrome (Pt1), in which primary B cells were impaired in activation due to delayed responses to CD40 signals [30]. This defect resulted in a lack of class switched antibodies with an attendant increase in infections. In vitro class switch recombination was restored if B cells received sustained signaling through CD40L expressed on 293 cells but not by activated CD4^+^ T cells. To recapitulate continuous CD40 signaling and determine whether the defect could be rescued and further analyzed, B cells were infected with Epstein Barr virus (EBV) and lymphoblastoid cell lines (LCLs) evaluated for maintenance or loss of the Pt1 phenotype in the context of sustained signals through the CD40 viral mimic, latent membrane protein (LMP)1. We found that the Pt1-LCLs and -LCL^tet^ cells (cells transformed with a mini-EBV vector expressing LMP1 from a tetracycline-inducible promoter [31]) failed to proliferate in response to CD40 signals however, there was growth in response to LMP1. Importantly, the Pt1-LCL^tet^ cells retained a subset of characteristics found in the primary B cells including reduced levels of surface CD23. These defects were not complemented by LMP1 and were directly linked to decreased c-Rel expression [32].

In this study, we have used the Pt1-LCL^tet^ cells to ask questions regarding cell proliferation and survival in EBV-transformed B cells that constitutively express low levels of c-Rel. Importantly, we found that proliferation and survival were impaired in these cells even in the context of continuous LMP1 expression. However, cell death was caspase-independent and highly consistent with autophagy followed by necrotic cell death. Additionally, ultrastructural imaging and gene expression profiling revealed that cells displayed a high degree of metabolic stress that was characterized by changes in the endoplasmic reticulum (ER) and the expression of inflammatory mediators. Notably, c-Rel was shown to be a negative regulator of caspase-4, a member of the inflammatory family of caspases involved in the regulation of the ER stress response, autophagy and cell survival. These results indicate that Pt1 cells experience a high degree of cellular stress that is linked to low c-Rel expression, reduced proliferation, and increased cell death. These findings provide important insights into the Pt1 defect and how EBV transformation may moderate the effect of low c-Rel expression on the viability of malignant B cells.

## Results

### Low c-Rel results in decreased c-myc expression

**Figure 1:**
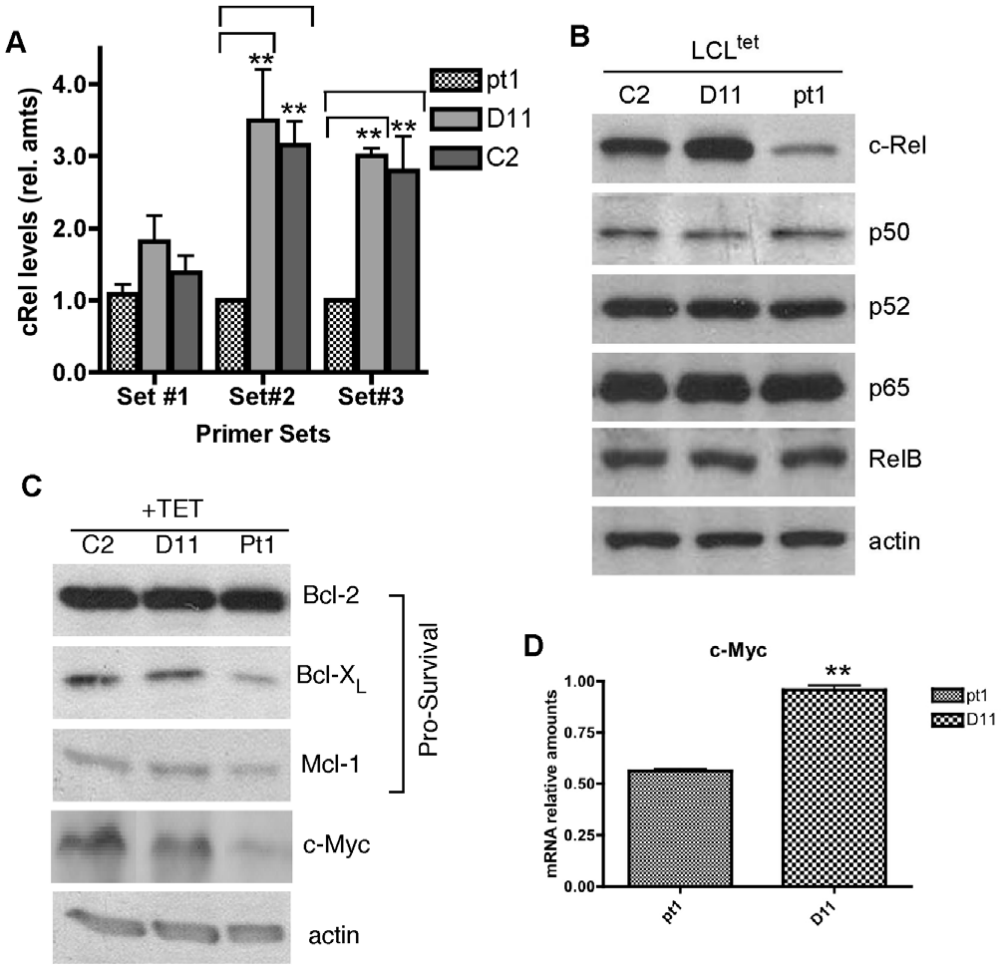
Decreased c-Rel in Pt1 cells is consistent with low c-Myc expression. (**A**) Relative amounts of c-Rel mRNA determined by quantitative real-time PCR (qRT-PCR) using three distinct sets of primers and cDNA generated from Pt1-, D11- and C2-LCL^tet^ cells. Set #1 (used in Lu, et. al. [32]), Set #2 (targeting the 3′ UTR) and Set #3 (targeting the 5′UTR). Numbers shown are the average and SEM from three independent experiments (** indicates a p<0.01). (**B**) 30 µg of total cell extract prepared from Pt1 and control (C2 and D11) cells grown in the presence of Tc were analyzed by immunoblotting for expression of NF-κB family members c-Rel, p50, p52, p65 and RelB. Actin is shown as a loading control. (**C**) Western Blot analysis showing the levels of pro-survival genes Bcl-2, Bcl-X_L_ and Mcl-1 as well as c-Myc in 30 µg of total cell extract prepared from Pt1- and control C2- and D11 -LCL^tet^ cells. Actin is shown as a loading control. (**D**) Analysis of c-myc expression using cDNA generated from RNA isolated from Pt1- and control D11-LCL^tet^ cells grown in Tc media and qRT-PCR. Numbers shown are the average and SEM of three independent experiments (** indicates a p<0.01).

Our previous analysis of c-Rel expression in Pt1 and control cells suggested that reduced c-Rel expression was due to translational or post-translational differences [32]. However, additional analyses using multiple primer pairs that targeted both the 5′ and 3′untranslated regions revealed that reduced c-Rel corresponded to a transcriptional defect that resulted in an approximate 3.5-fold decrease in RNA (Figure 1A). Examination of NF-κB subunit expression in Pt1 cells confirmed that c-Rel was selectively decreased and that all other Rel/NF-κB family members were expressed at control levels (Figure 1B). We also assayed the expression of proteins involved in cell survival and proliferation, which are known to be direct or indirect targets of c-Rel [29], [33]. As shown in Figures 1C and 1D, no consistent differences were detected in Bcl-2 and Mcl-1 expression between Pt1 and control cells whereas Bcl-xL was found to be slightly lower. In contrast c-myc expression at both the protein and RNA levels was clearly reduced, suggesting defects in cell survival and proliferation.

### The Pt1 cells have defects in proliferation

**Figure 2:**
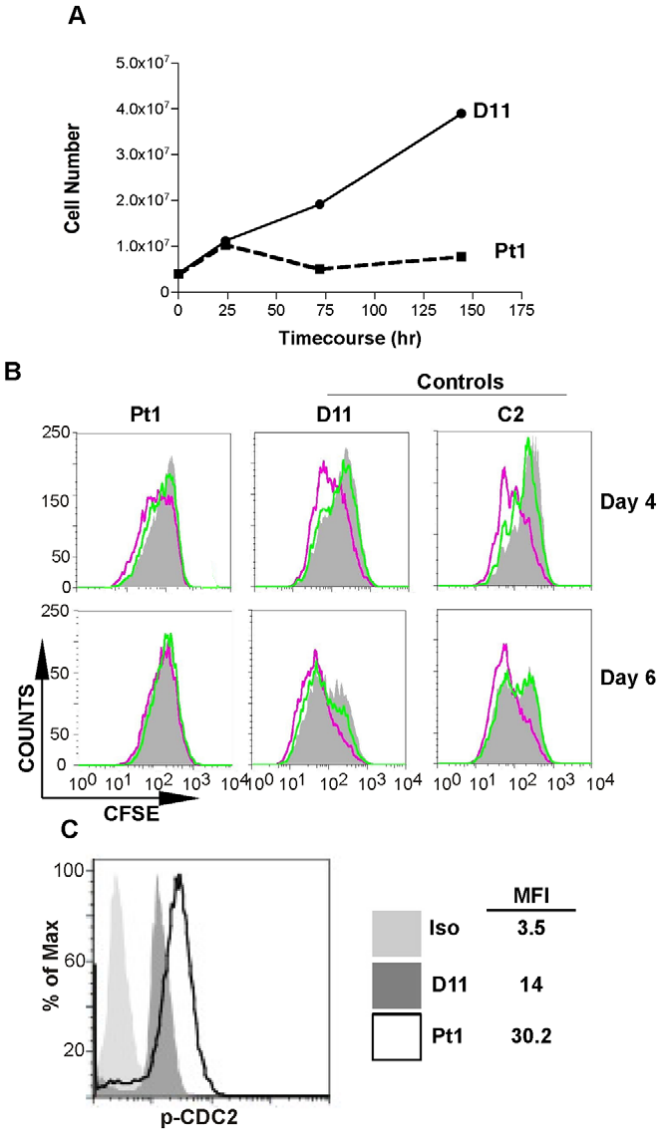
Pt1 cells have reduced proliferation in response to CD40 or LMP1 signaling. (**A**) Pt1- (dashed line) and control D11-LCL^tet^ cells (solid line) grown in the absence of Tc for 5 days were plated at 1×10^6^ cells/mL in Tc media and cultured in parallel for 6 days. Cells were withdrawn at days 1, 3 and 6 cells and counted using Trypan-Blue exclusion to determine the total number of viable cells. (**B**) 1×10^6^ Pt1- and control D11- and C2-LCL^tet^ cells were stained with CFSE and cultured in Tc media (pink line), absence of Tc (for 5 days prior to CFSE staining) (grey peak) or absence of Tc plus sCD40L (500 ng/mL) (green line) for 6 days. At day 4 (upper panel) and day 6 (lower panel) cells were analyzed for proliferation by flow cytometry. (**C**) Pt1- and control D11-LCL^tet^ cells (2×10^5^) grown in Tc media were analyzed for the expression of phosphorylated-CDC2 by intra-cellular staining. Numbers shown are the mean fluorescence intensity (MFI) of cells expressing high levels of phosphorylated CDC2. Graph is representative of three independent experiments.

To assess the degree that low c-Rel affected cell growth, cells were spun through a ficoll gradient to remove dead or dying cells from the population and 4×10^6^ viable cells were cultured in media containing tetracycline (Tc) to maintain LMP1 expression. While cultures of both Pt1 and control cells doubled during the initial 24 h period, only the control population continued to expand beyond this point (Figure 2A). The extent that the stationary phase reflected a proliferation defect was analyzed by monitoring CFSE incorporation in cells removed from Tc media for 5 days and re-plated in Tc media or media supplemented with soluble CD40 ligand (sCD40L) for 4 and 6 days. As expected, both control populations proliferated in response to LMP1 and sCD40L by day 4 and continued to do so through day 6 (Figure 2B, middle and right graphs). In contrast, the Pt1 population expressing LMP1 divided only after four days while no proliferation was detected at day six, whereas the cells stimulated with sCD40L showed little to no proliferation at either time point (upper and lower left graphs). Decreased proliferation was further confirmed by analyzing the phosphorylation level of CDC2, a protein that is functional during the G2/M checkpoint of the cell cycle. We observed that the mean fluorescence intensity (MFI) of the phosphorylated *inactive* form was approximately 2-fold greater in the Pt1 cells compared to D11 control cells indicating less cycling by the Pt1 population (Figure 2C).

### Cell death is occurring through a caspase-independent mechanism

**Figure 3:**
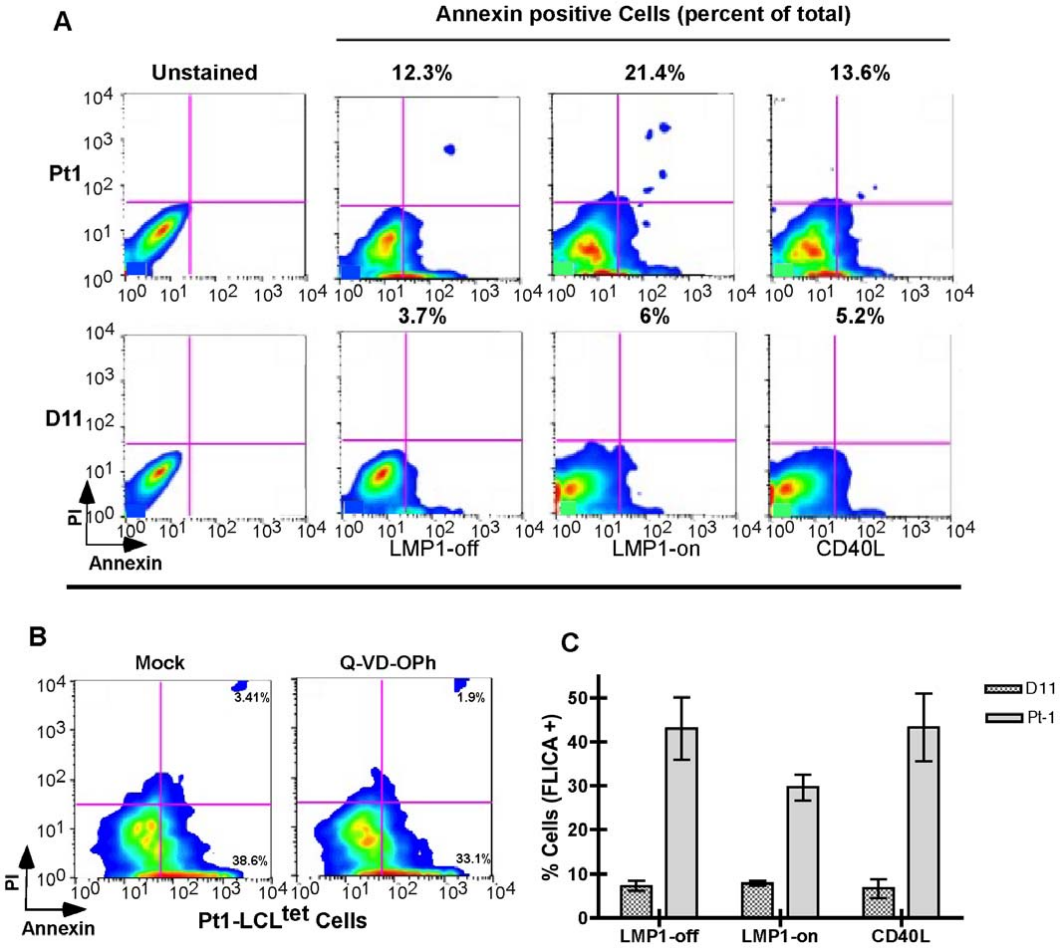
Increased cell death in the Pt1 cells is independent of caspase activity. (**A**) Analysis of cell viability in Pt1 and control populations grown in Tc media, absence of Tc (5 days) or absence of Tc+sCD40L (500 ng/mL) (24 h). 2×10^5^ cells were stained with Annexin-V and Propidium Iodide (PI) and analyzed by flow cytometry. Numbers shown are the percentage of Anexin-V^+^/PI^−^ cells (early cell death) plus Anexin-V^+^/PI^+^ (necrotic/late cell death). (**B**) 2×10^5^ Pt1 cells grown in Tc media were analyzed for cell death by flow cytometry 24 h post-treatment with 5 µM Q-VD-OPh. Numbers shown are the percentage of Anexin-V^+^/PI^−^ cells (early cell death) and Anexin-V^+^/PI^+^ (necrotic/late cell death). (**C**) Analysis of total levels of active caspases in Pt1 and control D11 cells grown in Tc media, absence of Tc (5 days) or absence of Tc+500 ng/ml sCD40L for 24 h. 2×10^5^ cells were incubated at 37°C for 2 h with polycaspase red-FLICA and analyzed by flow cytometry. Numbers shown are the average and SEM from three independent experiments.

Deletion of c-myc in activated primary B cells results in a severe loss of proliferative capacity [34] whereas down regulation of c-myc in several tumor lines results in both loss of proliferation and increased in cell death [35], [36], [37], [38]. Based on these observations and on our finding that c-Myc is down regulated in the Pt1 cells, we analyzed cells by Annexin V and propidium iodide (PI) staining for evidence of early- (Annexin V^+^, PI^−^) and late-stage (Annexin V^+^, PI^+^) cell death. We found that induction of LMP1 expression in the Pt1 cells resulted in an approximate 2-fold increase in the Annexin V positive population over cells cultured without LMP1. However, in agreement with the lack of proliferation in response to CD40, there was no significant increase in cell death upon stimulation with sCD40L (Figure 3A, upper panels). In contrast, the level of ongoing cell death in the control population was approximately 3-fold lower under all conditions (lower panels) suggesting that the Pt1 cells were more susceptible to cell death independent of CD40 or LMP1 signals.

To determine whether cell death was occurring through apoptosis, a broad-spectrum caspase inhibitor, Q-VD-OPh was added to Pt1 cells that had been cultured for several days in the presence of Tc media and proliferating at a very limited rate (see Figure 2A). Surprisingly, 24 h after addition of the inhibitor there was little to no change in the overall percentage of Annexin V positive cells suggesting that caspase activity was unlinked to cell death (Figure 3B). Additional hallmarks of apoptosis, including both PARP and caspase-3 cleavage, as well as increased expression of pro-apoptotic proteins Bax and Bad, were absent in Pt1 and control cells following growth with or without LMP1 (Figure S1). However, when the overall level of caspase activity was measured using sulforhodamine-FLICA, a fluorescent cell-permeable inhibitor that reacts with the active site of all caspases [39], it was clear that caspase activity was unusually high in Pt1 cells (Figure 3C). These findings indicated that the high level of caspase activity and poor viability of the Pt1 cells appeared linked to cell death exclusive of apoptosis.

### Pt1-LCL^tet^ cells display morphological characteristics of cellular stress

**Figure 4:**
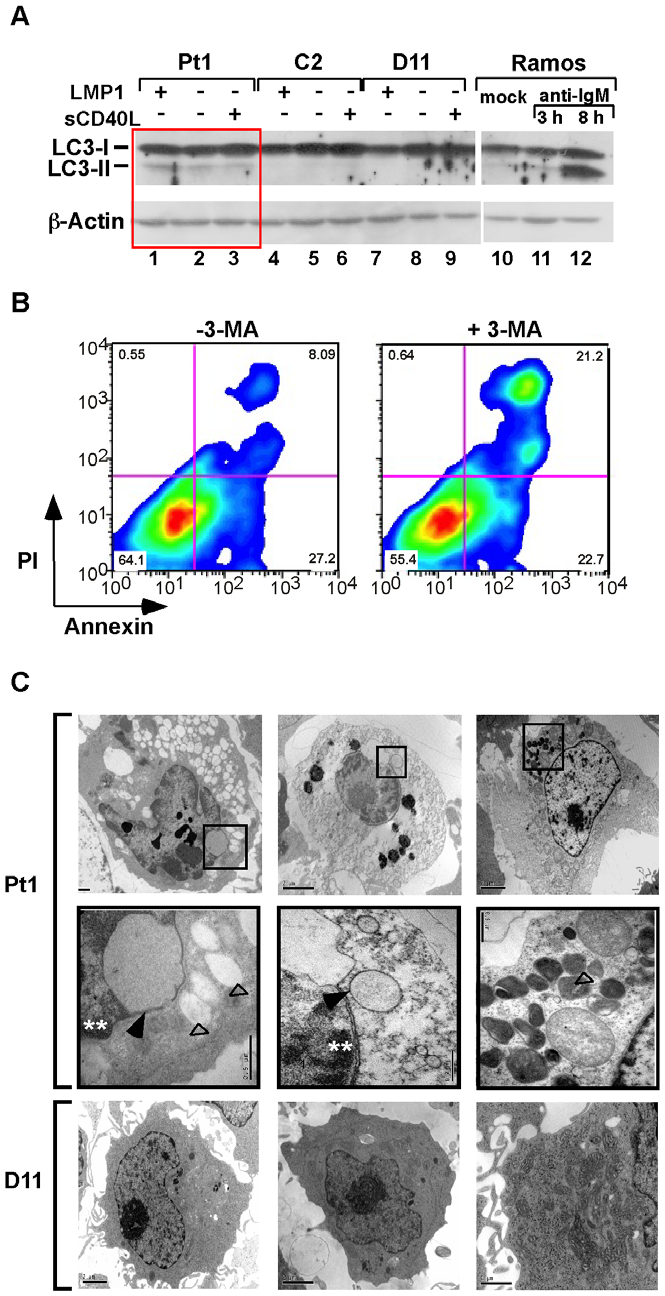
Pt1 cells display characteristic features of autophagy, ER-stress and necrosis. (**A**) LC3 expression in Pt1 and control (D11 and C2) cells. 30 µg of total cell extract isolated from Pt1 and control cells grown in Tc media, in the absence of Tc (5 days) or absence of Tc plus sCD40L (500 ng/mL) for 24 h was analyzed by immunoblotting using anti-LC3 antibodies. The Pt1 lanes are delineated with a red box to highlight the presence of LC3-II. Total cell extract (6 µg/mL) prepared from anti-IgM stimulated Ramos cells is shown as a positive control for LC3-II expression and actin is shown as a loading control. (**B**) 2×10^5^ Pt1-LCL^tet^ cells grown in Tc media were treated with 10 µM of the autophagy inhibitor 3-MA (right panel) or DMSO (left panel), stained with Annexin-V and Propidium Iodide (PI) and analyzed by flow cytometry. (**C**) Pt1- (upper and middle panels) and control D11-LCL^tet^ cells (lower panel) grown in Tc media were analyzed by transmission electron microscopy. Areas of high electron density (asterisks), dilations of the ER (open triangles) or autophagosomes (arrows) are observed only in the Pt1 cells and not in the control cells. Images in the middle panels are magnifications of the selected area (open square) shown on upper panel.

We next questioned whether autophagy was initiated in Pt1 cells as a pathway to enhance survival since it has been previously shown that under conditions of stress, cells defective in apoptosis will often activate autophagy as a cell survival mechanism [40], [41]. We initially assayed the increase in LC3-II expression, the lipodated product of LC3-I that is a marker for autophagy [42], [43] and found that Pt1 cells expressed a higher level of LC3-II relative to control cells under all conditions of culture (Figure 4A). Additionally, when cells were incubated with the PI3K inhibitor 3-Methyladenine (3-MA), that effectively prevents autophagosome formation, approximately a 3-fold increase in necrotic cell death (Annexin V^+^PI^+^) was detected with no significant change in early cell death (Annexin V^+^PI^−^) (Figure 4B). We interpret these findings to suggest that autophagy is an ongoing process in the Pt1 cells that occurs in the presence or absence of signals through CD40 or LMP1. Also, inhibition of autophagy results in a higher number of cells being rapidly shunted into a pathway leading to necrotic cell death.

To extend these findings and identify structural indications of autophagy, Transmission Electron Microscopy (TEM) was carried out using Pt1 and control D11 cells grown in Tc media for greater than 7 days (with LMP1 signaling). Autophagosomes were clearly present in the cytoplasm of Pt1 cells in a number of images (Figure 4C, see closed arrows). However, the number was more limited than expected and the more striking picture was one of extensive cellular stress and cell death (upper and middle panels). Specifically, the majority of cells contained a high number of translucent vacuoles in the cytoplasm that were consistent with endoplasmic reticulum (ER) dilations (see open triangles). In addition, there were many fewer mitochondria compared to control cells and a number of Pt1 cells displayed distortions in the nuclear membrane and electron-dense material in the nuclei (see asterisks). These images were in clear contrast to the control cells that displayed no outward signs of cellular stress or autophagy (lower panel). Together, these results are consistent with c-Rel-dependent defects in growth and proliferation resulting in an increased level of cellular and/or ER stress. Under these conditions autophagy appears to be activated as a survival strategy but is insufficient over time to compensate for the initiating stress and as a consequence cell death occurs through an apoptosis-independent pathway.

### Genes involved in ER and metabolic stress are activated in Pt1 cells

To understand how c-Rel may play a role in the phenotypic changes observed in the Pt1 cells we utilized microarray analysis to identify targets that were either co-modulated with c-Rel or potentially linked to both c-Rel expression and cellular or ER stress pathways. We assessed gene expression differences in the Pt1 cells following continuous growth in Tc media (greater than 7 days) against two control lines that showed differences in Ig expression and growth characteristics in order to highlight differences that were beyond normal phenotypic variation of immortalized LCLs [44], [45]. Of the 27,000 genes assessed, 764 (p = 0.05) displayed differential expression of 1.6-fold or higher. Importantly, we were able to show that both the expression of c-Rel and CD23 (*FCER2*), which had been previously identified as a *bona fide* c-Rel target, were decreased on the array [32]. However, there was no difference in the expression of pro-survival genes including Bcl-xL and thus the difference observed at the protein level was likely independent of a direct, c-Rel-specific affect on transcription (see Figure 1C). In contrast, a number of c-Rel regulated genes with roles in transcriptional regulation, cell structure, signaling and motility were found to be downregulated. In contrast, several other genes both related to c-Rel and the inflammatory response were upregulated compared to controls (Table 1).

**Table 1:** c-Rel-associated genes.

Gene name	Function	Fold change^1^	Relationship to c-Rel
FOXO3	Transcription factor	↓3.2	Transcriptional target
EGR2	Transcription factor	↓3.7	”
PPPIR15-A	Phosphatase	↓2.2	”
VIM	matrix protein	↓4.4	”
FCER2 (CD23)	Surface receptor	↓2.5	”
Myc	Cell-cycle regulator	↓2.1	”
IL1B	Inflammatory cytokine	↑7.9	”
CXCL10	Chemokine	↑5.7	”
TNFSF13C	Surface receptor	↑1.6	”
Bcl-xL	Pro-survival	N.C.	”
Mcl-1	Pro-survival	N.C.	”
Bfl-1/A1	Pro-survival	N.C.	”
IL1A	Inflammatory cytokine	↓2.9	c-Rel Nuclear translocation
HSPA1A	Protein folding	↑2.2	Protein-protein interaction
HSPA6	Protein folding	↑2.3	”

1 Arrows indicate the fold change difference in comparing the average expression of Pt1 genes (two duplicate samples) to duplicate arrays of two different control lymphoblastoid cell lines (four samples total). N.C. indicates “no change”.

In analyzing the overall changes in the Pt1 transcriptome we confirmed that a number of genes that mapped to metabolic/ER stress and autophagy pathways were differentially expressed (Table 2). Specifically, there was an increase in several heat shock genes as well as XBP1, a regulatory factor involved in ER stress. Ingenuity Pathway Analysis (IPA) was performed and pathways involving cell structure, motility and inflammation were found to be significantly different (data not shown). We were particularly interested in the changes relating to inflammation, which included highly expressed MMP7, IL-1β, IL-1βR, STAT3, TNFR, Cox-2 and others (Table 2). These data suggested that ongoing cell death and reduced proliferation were linked to autophagy, ER stress and a heightened inflammatory response in the Pt1 cells.

**Table 2:** Identified pathways and associated genes differentially expressed in pt1-LCL^tet^ cells^1^.

ER/metabolic stress	Function	Fold change
IEX-1 (IER3)	NF-κB regulator	↑4.5
HSPA6	Protein folding-heat shock	↑2.3
HSPA7	Protein folding-heat shock	↑2.0
Caspase-4	Cysteine protease	↑1.4
SDF2L1	O-Mannosylation	↑1.6
XBP1	Transcription factor	↑1.6
PDIA6	Isomerase	↑1.8
ATF5	Transcription factor	↑2.5
P62 (SQSTM1)	Signaling adaptor	↑1.6
PTP1B	Phosphatase	↑1.4
**Autophagy**		
DRAM	Lysosomal protein	↓1.6
ATG3	Protein ubiquitination	N. C.
ATG7	Protein ubiquitination	N. C.
ATG4A	Protease	N. C.
ATG4B	Protease	N. C.
GABARAP (ATG8A)	Receptor Binding protein	N. C.
**Inflammation**		
MMP7	Metalloproteinase	↑83.0
IL1B	Cytokine	↑7.8
IL1BR	Cytokine receptor	↑6.7
Stat3	Transcription Factor	↑1.9
CD28	Co-stimulatory receptor	↑2.3
CD19	Co-stimulatory receptor	↑3.5
CCL5	Chemokine	↑1.6
CCR7	Chemokine receptor	↑2.4

1 IPA on genes that were differentially expressed by a minimum of 1.4-fold. These represent only a subset of total identified genes. Arrows indicate positive and negative fold increases and N. C. indicates “no change”.

### Caspase-4 is upregulated in Pt1 cells

**Figure 5:**
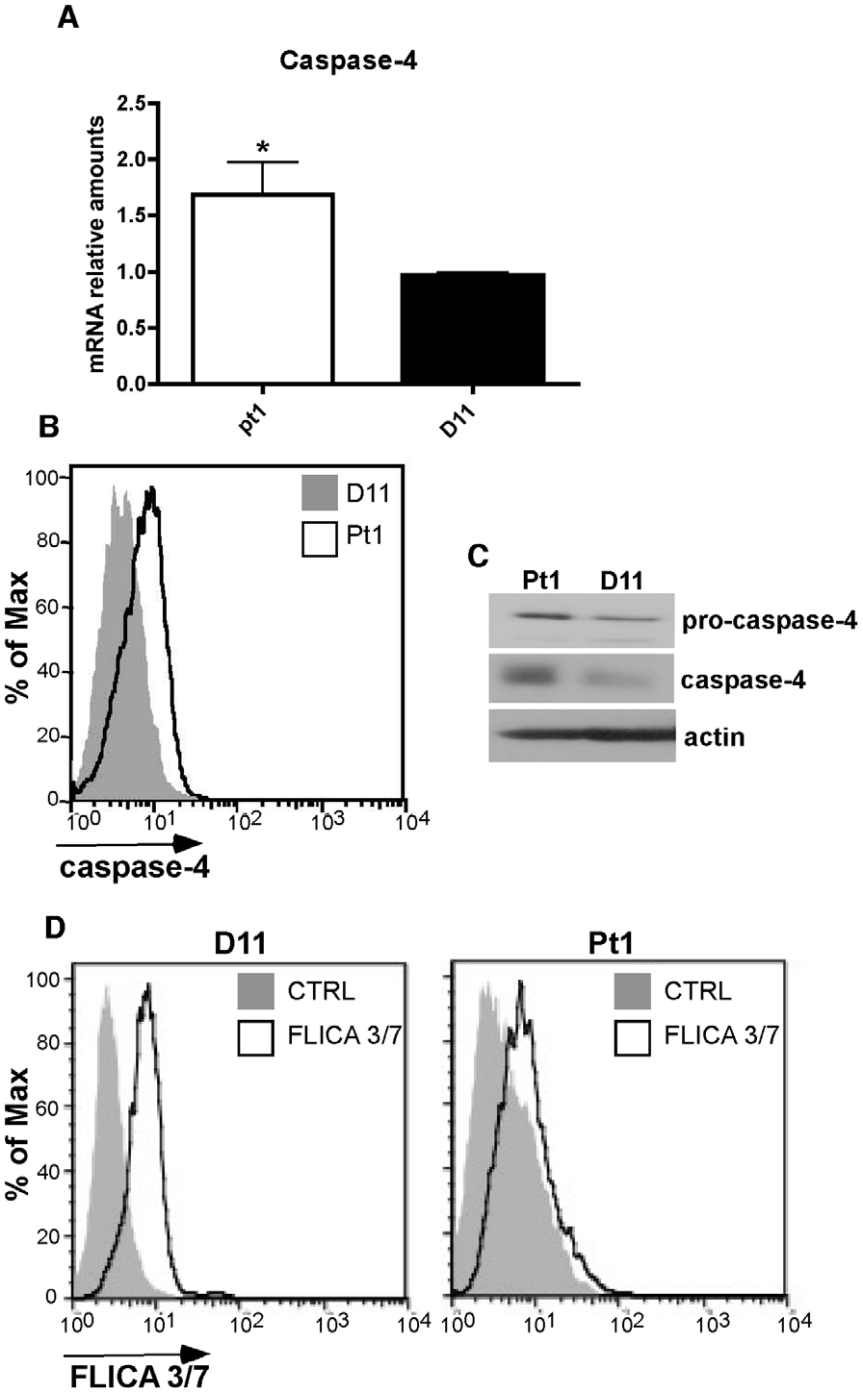
Caspase-4 is upregulated in Pt1 cells. (**A**) QRT-PCR analysis of caspase-4 expression in Pt1 and D11 control- LCL^tet^ cells. Numbers shown are the mean and SEM of three independent experiments (* indicates a p≤0.05) (**B**) Intra-cellular staining and flow cytometry analysis of total caspase-4 expression in 2×10^5^ Pt1 and D11 control-LCL^tet^ cells. (**C**) Western blot analysis showing the expression of the pro-caspase and active caspase-4 in 30 µg of total cell extract from Pt1 and D11 control LCL^tet^ cells. Actin is shown as a loading control. (**D**) Analysis of caspase 3 and 7 in control D11 (left) and Pt1 cells (right) following incubation with flica-specific for the active sites of these caspases. Histograms are representative of three independent experiments.

In support of our biochemical studies, we failed to observed transcriptional changes in factors relating either to the intrinsic or extrinsic apoptotic pathways (data not shown). However, we did note an increase in the expression of caspase-4 an ER-resident inflammatory caspase, which is a member of the interleukin-1β-coverting enzyme subfamily (reviewed in [46]). The expression of caspase-4 is modulated by interferons and induced in response to ER stress although few downstream target genes have been identified [47], [48], [49]. To determine whether caspase-4 was responsible for the increase in caspase activity present in Pt1 cells, we first confirmed that it was upregulated at the RNA level by qRT-PCR (Figure 5A). Additionally, intracellular staining revealed a higher level of total caspase-4 expression in Pt1 cells compared to control D11 cells (Figure 5B) and this increase was confirmed by Western blotting that showed a marked increase both in the pro and active forms of caspase-4 (Figure 5C). To rule out the possibility that the overall increase in caspase activity reflected an increase in the active forms of effector caspases, cells were incubated with a fluorescent competitor that binds specifically to the active sites of caspases-3 and -7 and no difference in binding was observed between control D11 and Pt1 cells (Figure 5D). Selective elevation of caspase-1 activity in Pt1 cells was similarly ruled out (data not shown). Thus, the absence of an increase in the active forms of caspases-3 and -7 as well as caspase-1 is highly consistent with the elevated caspase activity in Pt1 cells being an outcome of increased caspase-4 activation.

### Caspase-4 is regulated by c-Rel

**Figure 6:**
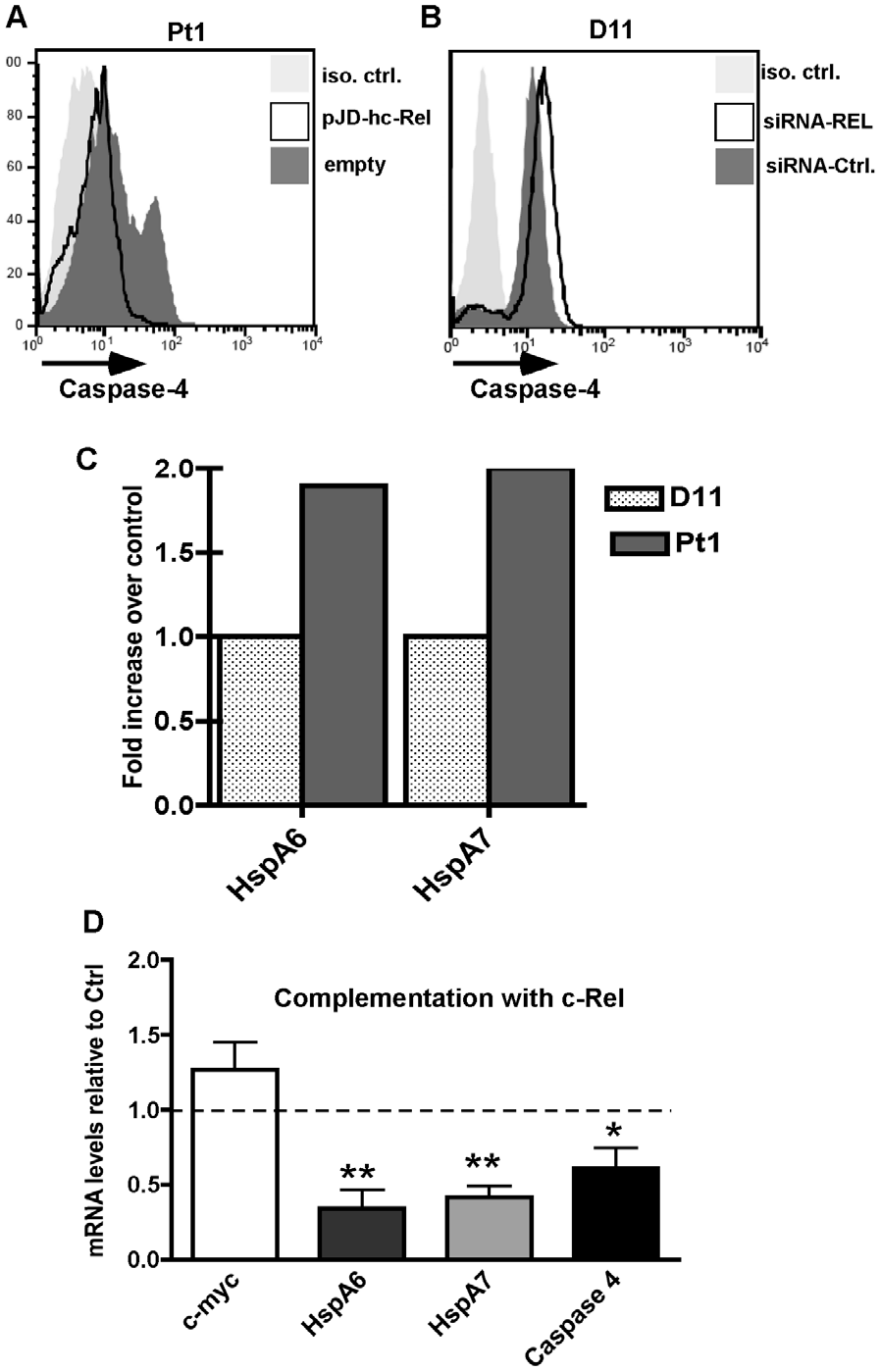
c-Rel is a negative regulator of caspase-4 in EBV lymphoblastoid cells. (A) Intra-cellular staining and flow cytometry analysis of total caspase-4 expression in 1×10^6^ Pt1-LCL^tet^ cells 48 h after cells were transiently transfected with either 20 mg of pJD-hc-Rel (open peak) or empty vector (dark grey peak). Histograms are representative of three independent experiments. (**B**) Analysis of caspase-4 expression in D11 control cells 48 h after cells were transiently transfected with 1.3 mM siREL (open peak) or control sequence (dark grey peak). In both panels, the isotype control is shown as a light grey peak. (**C**) QRT-PCR of HspA6 and HspA7 in cDNA generated from control C2-, D11- and Pt1-LCL^tet^ cells grown in Tc media. Control levels were averaged and set to “1”. (**D**) QRT-PCR of cDNA generated from Pt1-LCL^tet^ cells 48 h after transient transfection with 20 µg of pJD-hc-Rel. Results are the average of three independent experiments and show the change in expression relative to the cells transfected with a control vector (* indicates a p≤0.05; ** indicates a p≤0.01).

To establish whether low c-Rel was responsible for elevated caspase-4 activity we used transient transfection to increase c-Rel levels in Pt1 cells. However, due to the fragile state of the Pt1 population very few viable cells remained after transfection making biochemical analysis impossible. Expression was thus assessed using intracellular staining and gating on the viable population. In multiple experiments we observed a marked decrease in caspase-4 specifically in the c-Rel transfected population confirming a role for c-Rel as a negative regulator of caspase-4 expression (Figure 6A). We next asked whether down regulating c-Rel would result in an increase in caspase-4 expression in control cells. Control D11 cells were transfected with siRNA against c-Rel (siRel) or against a control sequence (siCTRL) and 48 h post-transfection caspase-4 expression was monitored using intracellular staining. The results of multiple transfections revealed a reproducible increase in caspase-4 expression in cells transfected with siRel (Figure 6B). The difference in the overall change relatively to the over-expression studies is most likely a consequence of the degree of downregulation of c-Rel protein, which was approximately 50% of wildtype levels (data not shown).

To extend these findings and ask whether increasing c-Rel would have an effect on the expression of genes involved in ER stress, we analyzed two heat shock proteins, HspA6 and HspA7, whose expression was upregulated on the microarray and further confirmed by real time PCR (Figure 6C). To this end, RNA was isolated from Pt1 cells 48 h after transfection with either the c-Rel-expressing plasmid or empty vector. We found that the levels of caspase-4, HspA6, and HspA7 RNA were significantly down regulated following transient expression of c-Rel. Also, we observed a small, but reproducible increase in c-myc expression (Figure 6D). Thus, the expression of c-Rel is linked to the rapid modulation of genes relating to both cell cycle regulation and ER stress.

## Discussion

In this report we demonstrate that low c-Rel expression in an EBV-transformed B cell line is coincident with decreased proliferation and reduced cell survival. However, unlike previous work showing that decreased NF-κB levels in EBV transformed LCLs leads to apoptosis [50], [51], our findings suggest that EBV modulates cellular responses to low c-Rel by decreasing viability through a process dependent on autophagy and necrotic cell death. Whereas we found that viable Pt1-LCL^tet^ cells respond to LMP1 signals and proliferate at wildtype levels for approximately 24 h, beyond this initial time frame, these low c-Rel-expressing cells undergo cell death at a higher rate than cells not receiving proliferative signals. This data suggested that proliferation and cell death are intricately linked and most likely reflect the fact that the cells are becoming increasingly metabolically stressed. Although *c-rel* deficiency has been shown to result in proliferation defects [9], [14], [15] B cells heterozygous for *c-rel* retained proliferative potential that was intermediate to wildtype and homozygous deleted cells suggesting that overall gene dosage is important for optimal c-Rel function [15]. In this regard, a low level of proliferation in Pt1 cells is consistent with c-Rel expression being low but not completely absent.

Our results showing that the Pt1 cells fail to undergo apoptosis in response to low c-Rel is highly distinct from previous experiments that reported increased growth arrest and apoptosis in LCLs with inhibited NF-κB activity [50], [51]. One study found that cell death lacked some of the hallmarks of programmed cell death [51] and although similar to our findings, the complete absence of an apoptotic signature in our work may reflect the fact that Pt1 cells have only a c-Rel deficiency and retain normal expression of the other NF-κB family members. Regulatory signals from these proteins may modulate the expression of pro-survival Bcl-2 family members by successfully preventing the induction of effector caspases (reviewed in [52]). It is interesting that CD40 signals were found to rescue mouse *c-rel* deficient B cells from apoptosis [15] and in our system the signals coming from LMP1 appear capable of substituting for this function. Notably, transcriptional profiling of B cells expressing an inducible LMP1 protein confirmed that a number of pro-survival genes are transcribed within 15 min of LMP1 expression [53], [54]. Our results demonstrate that reduced levels of c-Rel within the context of EBV transformation appear not to noticeably alter levels of known pro-survival targets of LMP1 (even though pro-apoptotic proteins, Bax and Bad are high) and this may in fact underlie the absence of apoptotic cell death. Thus, EBV infection appears to rescue cell death mediated by reduced levels of c-Rel but not c-Rel-dependent proliferation that is tightly associated with decreased levels of c-Myc.

A novel finding emerging from this work is that both caspase-4 activity and ER stress is closely linked to c-Rel expression. Whereas our understanding of caspase-4 regulation and potential downstream targets is still evolving, caspase-4 and its putative mouse homolog caspase-12 have been reported to directly connect ER stress to apoptosis. Activation of these inflammatory caspases initiates a cascade involving caspase-8 and -3 that is independent of mitochondrial involvement [47], [55]. However, in the Pt1 cells, caspase-4 fails to initiate apoptosis most likely because of the constant expression of Bcl-2 family members and as a default undergo autophagy followed by necrotic cell death. Notably, there is strong precedent for cells that are defective in both apoptosis and autophagy to respond to stress by activating an inflammatory program of gene expression and being shunted to a pathway of necrotic cell death (reviewed in [41], [56]). In particular, cells that are both *beclin* and *atg5* deficient are metabolically stressed and activate autophagy as a means of increasing survivability [57].

Overall these results expand our understanding of the relationship between c-Rel and LMP1, in particular how the co-expression of these two genes affect B cell viability. In many cells, reducing c-Rel expression would lead to arrested cell growth and apoptosis via the loss of pro-survival gene expression. However, if in fact the cell is associated with persistent LMP1 expression such as in post-transplant proliferative disease (PTPD), Hodgkin's lymphoma or nasopharyngeal carcinoma, reducing c-Rel would still lead to arrested proliferation but also lead to increased ER stress and autophagy. The failure of autophagy to restore cellular integrity would ultimately result in death by necrosis. Thus, targeting c-Rel may in fact be sufficient to limit proliferation and force EBV-associated tumors or -infected cells to engage an autophagic program in response to metabolic stress that ultimately leads to cell death by necrosis.

## Materials and Methods

### Antibodies and Cell Lines

The following polyclonal antibodies were obtained from Santa Cruz Biotechnology: rabbit polyclonal anti-c-Rel, rabbit polyclonal anti-c-Myc, goat polyclonal anti caspase-4, rabbit polyclonal anti-p65, goat polyclonal anti-p50, rabbit polyclonal anti-p52, rabbit polyclonal anti-RelB, and goat polyclonal anti-actin. Rabbit polyclonal anti-LC3 was a gift from Dr. Victor Jin, UMDNJ. Anti-S6 mAb, rabbit polyclonal anti-caspase 4 and rabbit polyclonal anti-phospho-CDC2 were purchased from Cell Signaling. Rabbit anti-Bax and anti-Bak polyclonal antisera were obtained from NeoMarkers. Mouse anti-Bcl-2 mAb, anti-Bcl-xL rabbit polyclonal anti-Mcl-1 rabbit polyclonal antisera were obtained from Ancell, LabVision and Chemicon respectively. The mini-EBV transformed (LCL^tet^) primary B cells from Pt1 and control healthy donors (C2 and D11) have been previously described [58]. These lines were originally generated following appropriate written consent and assent of the participants and with the approval of the Institutional Review Board of Rutgers, The State University of New Jersey. LCL^tet^ cell lines were maintained in RPMI-complete (10% fetal bovine serum (FBS), 2% penicillin/streptomycin, 1% L-glutamine, 1% sodium pyruvate and 1× non-essential amino acids) supplemented with 1 µg/mL tetracycline (Tc) or soluble CD40L (sCD40L) at a concentration of 500 ng/mL (Peprotec) for indicated time periods.

### Preparation of Total cell extract and immunoblotting

Cells were collected and washed with phosphate-buffered saline (PBS) and then sedimented by centrifugation at 1200 rpm for 5 min. The cell pellet was resuspended in RIPA buffer (150 mM NaCl, 1% NP-40, 50 mM Tris-Cl pH7.5, 5 mM EDTA. 0.1%SDS, 1 mM PMSF, 1× Protease inhibitor cocktail (Roche), 1 mM Sodium ortho-vanadate and 5 mM NaFl) and incubated 15 min on ice with vigorous mixing. The extract was centrifuged at 13,000 rpm at 4°C for 10 min. Supernatant was collected and stored at −80°C until use. Immunoblotting was carried out exactly as previously described [32].

### RNA isolation and Real Time quantitative PCR

Total RNA was isolated using TRIZOL (Invitrogen). DNAse1 treated RNA was cleaned using the RNAEasy kit (Qiagen) following the manufacturer's instructions and 1 µg of RNA was used to synthesize cDNA. Quantitative PCR was performed using a StepOne Real Time PCR machine (Applied Biosystems) using buffer and conditions suggested by the manufacturer. Select primer sequences are listed in Table S1 and all other primers are available upon request.

PCR amplification for human actin was performed on each sample as a control. Relative differences between samples were determined by calculating DDCT (Applied Biosystems). The DDCT values were converted to fold differences compared with control by raising 2 to the -DDCT power (2-DDCT).

### Cell Growth and Proliferation Assays

To assess cell growth in the population, Pt1 and control cells were purified over ficoll to remove non-viable cells and 4×10^6^ cells were plated in Tc media. At day 1, 3 and 6 cells were again purified over ficoll and viable cells were counted following incubation with Trypan Blue. To analyze proliferation, 1×10^6^ viable (Trypan-blue negative) cells were washed and resuspended in PBS+0.1%BSA and incubated for 10 min at RT with 5 mM 5, 6 carboxyfluoroscein diacetate succinimidyl ester (CFSE) (Molecular Probes). After incubation, the cell suspension was brought to 2% FBS and further incubated at 37°C for 10 min followed by 2 washes with PBS+2% FBS. Cells were plated in a 1 ml final volume of RPMI-complete in the presence or absence of Tc or in the absence of Tc plus 500 ng/mL sCD40L. Stimulated cells were incubated at 37°C for up to 6 days. Cells were collected at different time points, fixed in PBS+4% para-formaldehyde and analyzed by flow cytometry.

### Intracellular Staining

Intracellular staining was performed using the eBioscience Intra cellular staining kit. Briefly, cells were incubated in fixing/permeabilization buffer followed by incubation with primary antibody. Cells were washed and then incubated with PE-conjugated secondary antibody and analyzed by flow cytometry.

### Cell death and caspase activity

2.0×10^5^ Pt1 or control-LCL^tet^ cells were treated with 5 µM QV-D-OPh (Calbiochem), 10 µM Z-LEVD-fmk (BioVision) or with DMSO for 24 h and analyzed for cell death using the Annexin-V apoptosis detection Kit (Santa Cruz) following manufacturer's instructions. For autophagy inhibition, 2.0×10^5^ Pt1 cells were treated with 10 mM 3-Methyladenine (3-MA) (Fluka) or DMSO for 12 h and analyzed for cell death as previously described. Caspase activity was analyzed using the Policaspase Red FLICA kit (Immunochemistry Technologies). Briefly, 2.0×10^5^ Pt1- or control-LCL^tet^ cells were treated with Sulforhodamine FLICA for 2 h at 37°C as per manufacturer's instructions and analyzed by flow cytometry.

### Transmission Electron Microscopy (TEM)

Pt1- or D11-LCL^tet^ cells grown in Tc media and centrifuged at low speed to retrieve only live cells were fixed in Karnovsky's fixative (formaldehyde, 4% v/v and glutaraldehyde, 1% v/v, in 0.1 M Millonig's phosphate buffer, pH 7.3) for 3 h and incubated in 1% osmium tetroxide for 1 h followed by dehydration in a graded ethanol series. Cells were embedded in Spurr's embedding media (Electron Microscopy Sciences) and sectioned with a diamond knife (Ultracut E ultramicrotome; AO Reichert, Austria). Thin sections were stained with a 5% (w/v) uranyl acetate solution in 50% EtOH for 15 min followed by a 0.5% (w/v) lead citrate solution in CO_2_-free, double-distilled water for 2 min. Electron micrographs were taken using a model JEM 100 CX transmission electron microscope (JEOL).

### Array data analysis

Gene expression analysis was carried out at the Sanford-Burnham Medical Research Institute's Bioinformatics Shared Resource facility. Total RNA samples were prepared in Trizol from Pt1 and control cells grown in Tc media and centrifuged at low speed to pellet only live cells. Analysis was carried out on a human-HT Beadchip Array (48K probes) using an Illumina Platform. Data were collected, and the initial analysis was performed using GeneSpring GX11 software (Agilent). Only the statistically significant data (*p*<0.05) were analyzed further. Pathway clustering of genes showing greater than a 1.4-fold difference between Pt1 and controls was carried out using Ingenuity Pathway Analysis (IPA).

### c-Rel Transfection

20 µg of plasmid pJDCMV19SVhc-Rel (pJD-hc-Rel) (a gift from C. Gelinas (UMDNJ)), pJDCMV19SVhc-empty or 2 µg of pMaxGFP to calculate transfection efficiency were transfected into between 1×10^6^ and 3×10^6^ Pt1-LCL^tet^ cells [58] using the Amaxa Nucleofactor Transfection System (Lonza) (Solution V, program X-001). 48 h post-transfection a percentage of the cells were harvested and analyzed for expression of caspase 4 by flow cytometry. The remaining cells were collected and total RNA was isolated and used to generate cDNA for quantitative real-time PCR analysis of multiple genes. Transfection efficiency was between 45% and 55% for each experiment.

### siRNA transfection

c-Rel or control siRNA (Santa Cruz) at a final concentration of 1.3 mM were transfected into control-LCL^tet^ cells using the Amaxa Nucleofactor Transfection System (Lonza) (Solution V, program X-001). 48 h post-transfection, cells were harvested and analyzed for caspase-4 expression by flow cytometry.

### Statistics

Mean with SE was calculated and data analyzed with Student paired and unpaired *t* tests to determine statistical significance of differences. Statistical analyses were performed using GraphPad Prism (GraphPad Software). Values of p<0.05 were accepted as statistically significant.

## Supporting Information

Figure S1 **Increased cell death in Pt1 cells does not correspond to enhanced PARP, or Caspase 3 cleavage.** 30 mg of total extracts from controls C2-, D11- and Pt1-LCL^tet^ cells grown either in the presence or absence of Tc were analyzed for the expression of (A) PARP, (B) the pro-and active forms of caspase 3 and (C) proapoptotic proteins Bax and Bad.
(TIF)

Table S1 **Primer Sequences used in qRT-PCR analysis.**
(DOC)
