# Supplementary figures and images for: c-Rel Deficiency Increases Caspase-4 Expression and Leads to ER Stress and Necrosis in EBV-Transformed Cells
Source: PLoS One. 2011 Oct 3;6(10):e25467. doi: 10.1371/journal.pone.0025467 (PMC3184984; doi:10.1371/journal.pone.0025467)

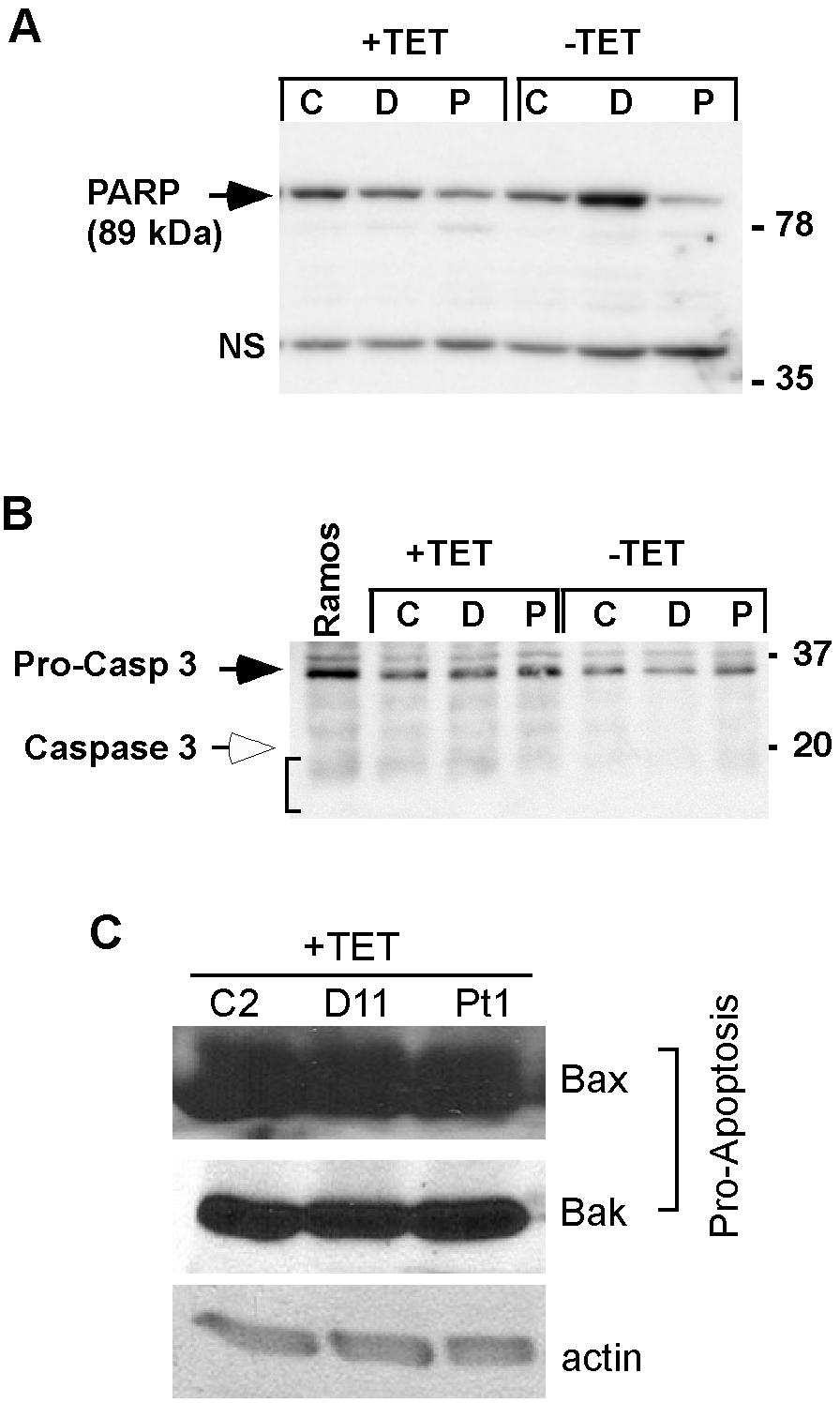

Supplement: Figure S1 — Increased cell death in Pt1 cells does not correspond to enhanced PARP, or Caspase 3 cleavage. 30 mg of total extracts from controls C2-, D11- and Pt1-LCLtet cells grown either in the presence or absence of Tc were analyzed for the expression of (A) PARP, (B) the pro-and active forms of caspase 3 and (C) proapoptotic proteins Bax and Bad. (TIF) [file pone.0025467.s001.tif]
