# Supplementary material for: c-Rel Deficiency Increases Caspase-4 Expression and Leads to ER Stress and Necrosis in EBV-Transformed Cells
Source: PLoS One. 2011 Oct 3;6(10):e25467. doi: 10.1371/journal.pone.0025467 (PMC3184984; doi:10.1371/journal.pone.0025467)
Supplement: Table S1 — Primer Sequences used in qRT-PCR analysis. (DOC) [file pone.0025467.s002.doc]

**Table S1.** **Primer Sequences used in qPCR**

| **Name** | **Sequence** |
| --- | --- |
| c-Rel#1(F)(692-713) | 5’-CGAACCCAATTTATGACAACCG-3’ |
| c-Rel#1(R)(1061-1038) | 5’-TTTTGTTTCTTTGCTTTATTGCCG-3’ |
| c-Rel#2(F)(1358-1389) | 5’-TCTCAAGTGGATTGTCACATCA-3’ |
| c-Rel#2(R)(1423-1404) | 5’-CCACTGATGACCAGCTTGAA-3’ |
| c-Rel#3(F)(98-117) | 5’-GGCCTCCTGACTGACTGACT-3’ |
| c-Rel#3(R)(229-208) | 5’-GCCTGGGTTGTTCAATTATCTC-3’ |
| c-Myc(F) | 5’-AGGGTCAAGTTGGACAGTGTC-3’ |
| c-Myc(R) | 5’-TCGTGCATTTTCGGTTGTTG-3’ |
| Caspase-4(F) | 5’-TTGCTTTCTGCTCTTCAACG-3’ |
| Caspase-4(R) | 5’-GTGTGATGAAGATAGAGCCCATT-3’ |
